# Death under Ether

**Published:** 1878-08

**Authors:** 


### Death under Ether.

A coal-porter, over fifty years of age, was admitted into the London Hospital for strangulated hernia last week. He was a well-developed man, and had been the subject of inguinal hernia more than three years, but till the last week had always been able to restrain it, and had never worn a truss. He had never experienced any special inconvenience from the rupture till four days before his admission to the hospital, when he was troubled with abdominal pain, complete obstruction of the bowels, and frequent vomiting, latterly becoming fæcal. His general health had been failing in an increasing degree during the last few years, so that he had sought medical advice. When admitted to hospital, he complained much of abdominal pain, and was unable to reduce his rupture. When examined, he presented a large scrotal hernia, somewhat tense. During the examination, he began vomiting fecal matter. Taxis having been used, but the symptoms remaining unrelieved, it was considered advisable to place the man under an anæsthetic. The house-surgeon accordingly administered ether, using not more than an ounce and a half in all. The patient came under the influence of the ether rapidly, and without difficulty or adverse symptoms. The local examination was then proceeded with. The patient's respirations were perfectly regular, and his pulse good, when, about six minutes after inhalation began, a sudden spasmodic inspiratory sound was heard, as if he were choking. His tongue was immediately drawn forward with the forceps; but respiration had ceased, although his pulse continued to beat for another half-minute. Silvester's artificial respiration was employed, but no spontaneous inspiratory effort followed. During the artificial respiration, scme fecal matter came up into the mouth. These efforts continued a quarter of an hour, but proved useless. At the *post-mortem* examination, the left ventricle was found contracted; but the heart appeared healthy. The lungs were extremely congested. There was fecal staining of the œsophagus and larynx, but no such matter had been drawn into the lungs. The liver was healthy. The kidneys were slightly granular, but not congested. The portion of small intestine which had been strangulated was about twenty inches in length, extremely congested, with commencing peritonitis upon its surface. It must be remembered that in such a case as this, where strangulation had existed four days and was attended with constant vomiting and a feeble circulation, there were many circumstances predisposing to death besides the administration of ether.—*Med. News.*

